# Assay Interference Causing Persistently Elevated Vancomycin Levels Leading to Treatment Failure and Fatal Outcome

**DOI:** 10.7759/cureus.61943

**Published:** 2024-06-08

**Authors:** Sindhu C Pokhriyal, Ernestine Faye S Tan, Parjanya K Bhatt, Ahmad Ali Khan, Muhammad N Pasha, Luckencia Pierre, Kalpana Panigrahi

**Affiliations:** 1 Internal Medicine, One Brooklyn Health-Interfaith Medical Center, Brooklyn, USA; 2 Medicine, B. J. Medical College, Ahmedabad, IND; 3 Pulmonary and Critical Care Medicine, One Brooklyn Health-Interfaith Medical Center, Brooklyn, USA

**Keywords:** staph aureus, staphylococcus aureus, treatment failure, antibiotic treatment failure, antibiotic treatment, antibiotic, cellulitis, assay interference, vancomycin

## Abstract

In patients receiving vancomycin therapy, serum drug levels are routinely monitored to ensure therapeutic dosing and minimize toxicity. In rare cases, vancomycin levels may be falsely or persistently elevated without any apparent cause. In this case report, we explore a rare case of persistently elevated vancomycin levels despite discontinuation of the drug for days.

This is a case of a 69-year-old female admitted for altered mental status secondary to sepsis from leg cellulitis. Antibiotic therapy included vancomycin. To ensure proper dosing, vancomycin trough levels were collected before the fourth dose, and the result showed a high value of 39 ug/ml. Vancomycin doses were adjusted as per the Bayesian dosing software, and the same remained to be in supratherapeutic levels. The patient eventually deteriorated, and due to persistently high vancomycin levels, the antibiotic regimen was switched to a different antibiotic. Despite normal renal functions, the vancomycin levels remained high, between 27 ug/ml and 32 ug/ml, even in the absence of any further doses. Subsequently, vancomycin serum concentration was determined by another method using high-performance liquid chromatography (HPLC). Blood cultures grew both coagulase-negative *Staphylococcus aureus* and *Achromobacter xylosoxidans*. Vancomycin levels remained high a week after discontinuation of the drug. Vancomycin by HPLC assay eventually showed that vancomycin was undetectable in the blood, but, unfortunately, the results came at a time when the patient had already expired.

In conclusion, clinicians should maintain a high level of suspicion if persistently higher vancomycin levels cannot be accounted for by renal function or other causes. In patients with persistently high vancomycin levels who continue to clinically deteriorate, it is crucial to consider that assay interference can result in inaccurately elevated vancomycin levels.

## Introduction

Vancomycin is a broad-spectrum glycopeptide antibiotic commonly used in the hospital setting for infections involving gram-positive bacteria, such as methicillin-resistant *Staphylococcus aureus*. Vancomycin has many potential side effects, such as ototoxicity, nephrotoxicity, hypotension, and infusion-related toxicities. Therefore, in patients receiving vancomycin therapy, serum drug levels are routinely monitored to ensure dosing remains therapeutic and below toxic levels. Monitoring is usually done by obtaining serum vancomycin trough or random vancomycin levels. In rare cases, serum vancomycin levels may be falsely or persistently elevated, presenting an inaccurate picture of the progress of therapy. 

In this report, we present the case of a patient who had persistently high levels of vancomycin despite a normal kidney function. The patient continued to have supratherapeutic serum vancomycin levels despite discontinuation of the drug for 10 days. This case aims to highlight the importance of clinical correlation of laboratory results in guiding treatment, especially when the patients are not improving despite elevated drug levels. 

## Case presentation

This is a case of a 69-year-old female with a past medical history of asthma, obesity, diabetes mellitus, hypertension, heart failure with reduced ejection fraction, left knee replacement, and chronic left leg ulcer. The patient presented with a six-day history of progressively worsening leg pain, decreased appetite, and fever. As per the son, the patient had a wound on the left leg for more than a year, which he regularly dresses, but recently the patient has been lost to follow-up with her wound care specialist. 

**Figure 1:**
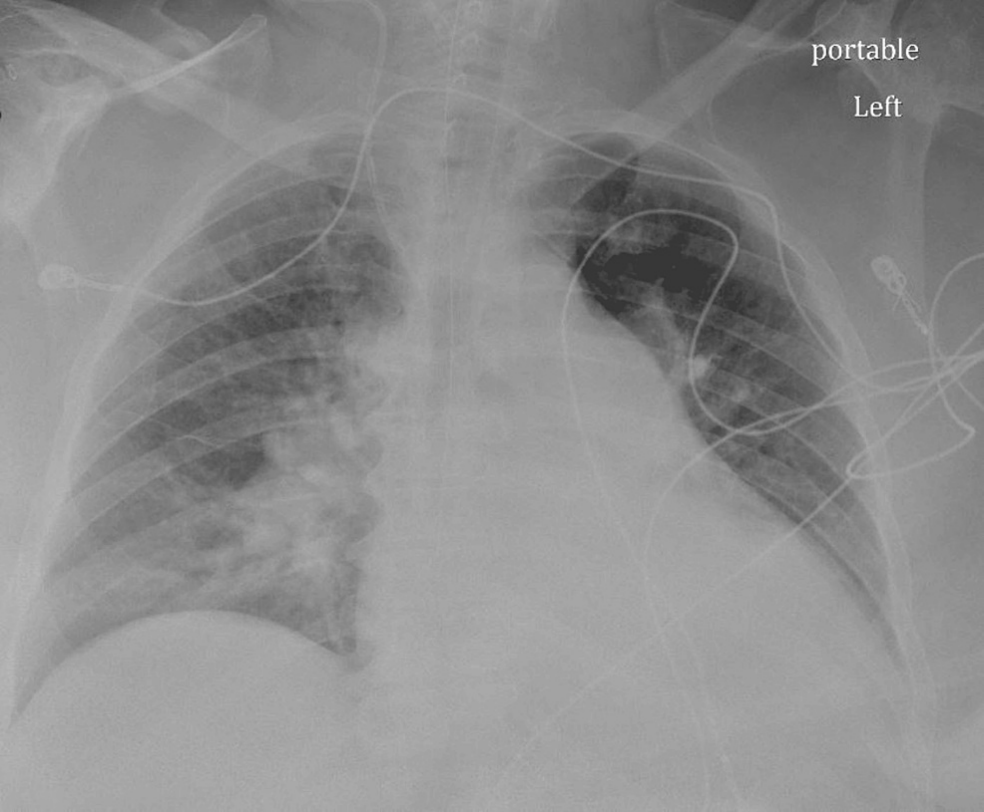
Chest X-ray on admission

**Figure 2:**
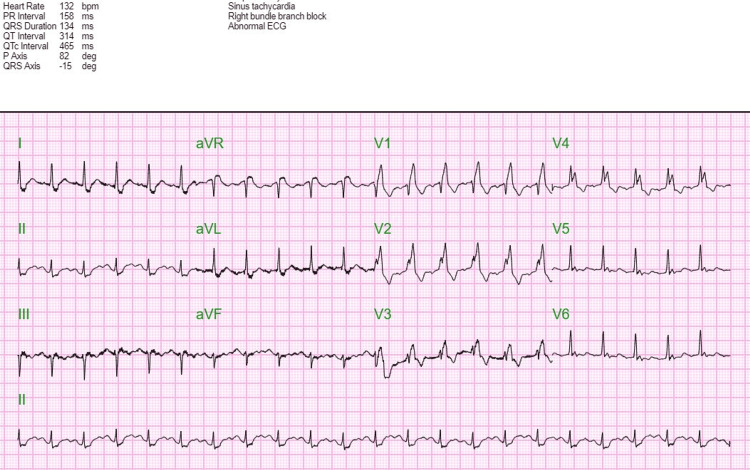
EKG on admission EKG: electrocardiogram

The patient was tachycardic and hypotensive on presentation, with hypoxic respiratory failure, metabolic encephalopathy, and sepsis due to left leg cellulitis. In addition, the patient had a positive COVID-19 polymerase chain reaction (PCR) test. As per the son, the patient did not exhibit any signs and symptoms of COVID-19 prior to admission, and the duration of infection was unknown. The patient was intubated due to severe respiratory distress, received bebtelovimab (monoclonal antibody) for COVID-19, and hydrated accordingly per sepsis protocol. Cultures were sent. The patient had allergies to clarithromycin, penicillins, levofloxacin, ciprofloxacin, and sulfamethoxazole-trimethoprim. Therefore, she was started on intravenous (IV) aztreonam, doxycycline, and vancomycin for sepsis. The patient was also noted to have bloody mucoid stools, for which metronidazole was added. Laboratory results were done, the results of which are outlined in Table 1. Chest X-ray showed mild cardiomegaly with no consolidation, but with heterogeneous opacities (Figure 1), and electrocardiogram (EKG) showed sinus tachycardia with right bundle branch block (Figure 2). Transtracheal aspiration (TTA) culture grew *Pseudomonas aeruginosa*, and inhaled tobramycin was added, for which the organism was sensitive (Table 2).

**Table 1:** Patient's laboratory findings on admission

Laboratory test	Normal range	Results
White blood cell	4.5-11.0 10x3/uL	14.1
Hemoglobin	11.0-15.0 g/dL	7.4
Hematocrit	35-46%	24.3
Mean corpuscular volume	80-100 fL	68.1
Platelets	130-400 10x3/uL	338
Blood urea nitrogen	9.8-20.1 mg/dL	64.3
Creatinine	0.57-1.11 mg/dL	0.83
Estimated glomerular filtration rate	≥90.0 mL/min/1.73 m^2^	76.3
Potassium	3.5-5.1 mmol/L	3.9
Sodium	133-145 meQ/L	132
Phosphorus	2.3-4.7 mg/dL	6.8
Magnesium	1.6-2.6 mg/dL	2.2
Calcium	8.4-10.5 mg/dL	8.1
Vitamin D	30-100 ng/mL	13
Prothrombin time	9.8-13.4 seconds	18.1
International normalized ratio	0.85-1.15 ratio	1.58
Troponin I	0.0-17 ng/L	8
Partial thromboplastin time	24.9-35.9 seconds	48.4
Brain natriuretic peptide	10.0-100.0 pg/mL	206

**Table 2:** Culture and sensitivity of tracheal aspirate (Pseudomonas aeruginosa)

Antibiotic	Sensitivity
Amikacin	Sensitive
Aztreonam	Resistant
Cefepime	Intermediate
Ceftazidime	Sensitive
Ciprofloxacin	Sensitive
Gentamicin	Intermediate
Imipenem	Intermediate
Levofloxacin	Intermediate
Piperacillin-tazobactam	Sensitive
Tobramycin	Sensitive

Before the fourth dose of vancomycin, trough levels were collected. Results showed a high value of 39 μg/mL. Vancomycin doses were adjusted as per the Bayesian dosing software. Despite normal renal functions, the vancomycin levels remained high over the next few days, ranging from 27 μg/mL to 32 μg/mL using particle-enhanced turbidimetric inhibition immunoassay, even in the absence of any further doses (Table 3). Subsequently, vancomycin serum concentration was sent by another method using high-performance liquid chromatography (HPLC). Repeat blood cultures grew both coagulase-negative *Staphylococcus aureus* and *Achromobacter xylosoxidans* (Table 4), and TTA continued to grow *Pseudomonas aeruginosa* that was resistant to aztreonam. A repeat chest X-ray showed worsening pneumonia despite treatment. Antibiotics were escalated to meropenem, and doxycycline was continued. Linezolid was added to the antibiotic regimen to cover for *Staphylococcus aureus*. The patient continued to be COVID-19 positive per PCR. Her blood pressure readings ranged from 72/42 to 109/56, for which she required multiple vasopressor support and hydrocortisone as adjunctive treatment for septic shock. The patient eventually deteriorated clinically, ultimately succumbing to severe sepsis from methicillin-resistant *Staphylococcus aureus, Pseudomonas aeruginosa, *and *Achromobacter xylosoxidans *bacteremia. Vancomycin by HPLC assay result eventually showed undetectable levels in the blood, but unfortunately, the results came after the patient had already passed. 

**Table 3:** Vancomycin level trends of the patient (in ug/mL)

Vancomycin level after initiation (trough)	Vancomycin level before discontinuation (trough)	Day 1 after discontinuation (trough)	Day 5 after discontinuation (random)	Day 7 after discontinuation (random)	Day 10 after discontinuation (random)
39	33.0	30.9	30.1	30.7	31.0

**Table 4:** Blood culture growth and sensitivity

Antibiotic	*Achromobacter xylosoxidans* spp. *denitrificans*	Staphylococcus aureus
Amikacin	Resistant	N/A
Amoxicillin-clavulanate	N/A	Resistant
Ampicillin	N/A	Resistant
Ampicillin-sulbactam	N/A	Resistant
Azithromycin	N/A	Resistant
Aztreonam	Resistant	N/A
Cefazolin	N/A	Resistant
Cefepime	Resistant	N/A
Ceftazidime	Resistant	N/A
Ceftriaxone	Resistant	N/A
Ciprofloxacin	Resistant	Resistant
Clindamycin	N/A	Resistant
Erythromycin	N/A	Resistant
Gentamicin	Resistant	Resistant
Levofloxacin	Sensitive	Resistant
Oxacillin	N/A	Resistant
Penicillin	N/A	Resistant
Piperacillin-tazobactam	Intermediate	N/A
Rifampin	N/A	Resistant
Tetracycline	Resistant	N/A
Tobramycin	Resistant	N/A
Trimethoprim+sulfamethoxazole	Sensitive	Resistant
Vancomycin	N/A	Sensitive

## Discussion

Methicillin-resistant *Staphylococcus aureus *is a significant pathogen that commonly affects critically ill patients, causing approximately 25.8% of all bacteremias, 10% of which eventually develop septic shock [1,2]. In a study by Blot and associates, methicillin-resistant *Staphylococcus aureus* bacteremia resulted in significantly more renal injuries, hemodynamic instabilities, and mortality rates than methicillin-sensitive *Staphylococcus aureus* bacteremia[3]. For these reasons, vancomycin remains the initial drug of choice in critically ill patients suspected to have gram-positive infection[4]. 

Vancomycin is a tricyclic glycopeptide which exerts bactericidal activity by binding to D-alanine and inhibiting P-phospholipid carrier and peptidoglycan synthase, thereby weakening the cell wall and causing cell death [5,6]. It is FDA-approved for both oral and IV administration. Its oral bioavailability is low, with about 55% protein binding, and it is widely distributed throughout bodily tissues and fluids, with the exception of the cerebral fluid in non-inflamed meninges. Vancomycin is primarily eliminated by glomerular filtration in the kidneys, with a biphasic elimination half-life of 4-6 hours in healthy adults with normal renal function[7]. Nephrotoxicity, hypotension, and anaphylactic reactions are some of the known side effects of IV vancomycin [8]. Drug committees in hospitals ensure proper and regulated usage of vancomycin. Pharmacists in the majority of healthcare facilities make sure the prescription is appropriate and that dosage levels are tracked in accordance with protocols [9].

**Figure 3:**
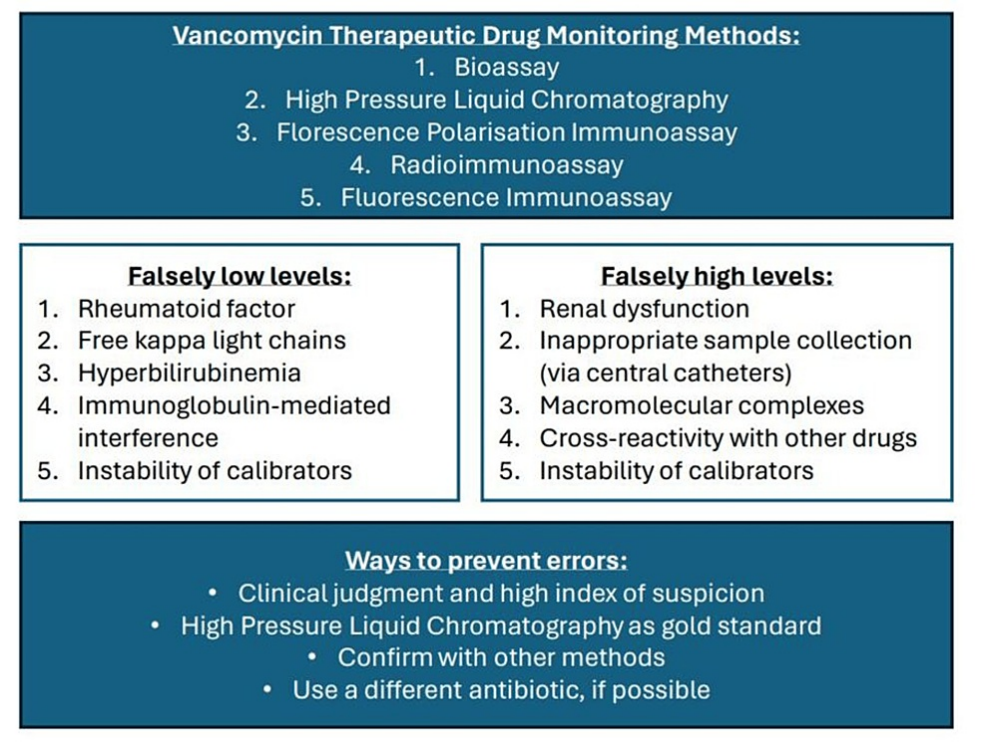
Factors that may affect vancomycin therapeutic drug monitoring EMR: electronic medical record

To date, the adjustment of vancomycin dosing remains a challenge. In patients receiving vancomycin treatment, the standard of practice is to ensure that the serum vancomycin levels remain in the therapeutic range to optimize effectiveness and minimize toxicity [10]. Once a steady state has been reached, the trough levels are typically measured one hour before every third or fourth dose is administered [7]. This is usually monitored through immunoassays[11]. However, some reports have demonstrated evidence of protein interferences in immunoassays, resulting in falsely or persistently elevated vancomycin levels [10-12]. Cases have also been reported of elevated vancomycin levels prior to receiving the drug or in the absence of vancomycin therapy [10,11]. Figure 3 outlines several factors that may falsely increase or decrease vancomycin levels[12-15]. 

Considering all these challenges, vancomycin administration has been individualized using area under curve (AUC)-guided methods such as equation-based and Bayesian-derived methodologies [13]. The Bayesian technique is based on Bayes' theorem, which estimates the vancomycin AUC value with minimal bias using a priori probability. This allows for the real-time provision of a dosing plan. The benefit of the Bayesian technique is that readings can be acquired 24-48 hours after the initial dose, instead of waiting until the vancomycin reaches a steady-state concentration. AUC estimates can be applied to all patients, including those who are obese, those who are critically unwell, pediatric patients, or those who have renal impairment. Additionally, this information helps with adjusting later dose schedules. At present, the suggested approach is to use the peak and trough vancomycin concentrations to estimate the Bayesian AUC. A 400-600 Bayesian-derived AUC is the goal in order to maximize drug efficacy and minimize the risk of acute kidney injury (AKI) [14]. The Bayesian-derived software is readily available and can be used to identify optimal dosing recommendations to achieve predefined AUC targets [16]. 

In this patient, immunoassay testing revealed unusually elevated vancomycin drug levels. In most cases, a serum trough level of 15-20 μg/mL is recommended for vancomycin in various complicated infectious diseases [16,17]. However, this patient had an initial vancomycin level of 39 μg/mL and persisted to have levels above 25 μg/mL despite normal renal functions and discontinuation of treatment, which did not correlate with the clinical picture. A similar case was reported by Tsoi and colleagues, which they have attributed as due to possible endogenous protein interference, as immunoassays are more susceptible to cross-reactivity[6]. Several studies also show erroneously elevated vancomycin levels detected from immunoassays as compared to levels determined from HPLC [10,12,18]. HPLC, as compared to immunoassays, is more reliable due to its higher sensitivity and selectivity [18]. It is also less susceptible to cross-reactions with vancomycin metabolites that could cause false-positive results in immunoassays [18].

In Figure 3, we listed five different methods to monitor vancomycin levels, which include bioassay, HPLC, fluorescence polarization immunoassay (FPIA), radioimmunoassay (RIA), and fluorescence immunoassay [18]. Several factors may also cause falsely high or falsely low vancomycin levels, which are listed in Figure 3 [12-14,18]. For future directions, such correspondence between clinical judgment and laboratory values can be minimized by proper training of the professionals, critical thinking skills, multidisciplinary approaches, use of gold standard HPLC for confirmation, and modified FPIA to prevent overestimation. New software tools are also in development to predict this phenomenon [12-14,18]. 

## Conclusions

The implementation of therapeutic drug monitoring is widely accepted and is a customary approach in the administration of vancomycin, with the primary objective of optimizing patient outcomes while minimizing the potential for toxicity. It is crucial to consider that assay interference can result in inaccurately elevated vancomycin levels. Clinicians should maintain a high level of suspicion if persistently higher vancomycin levels cannot be accounted for by renal function or other causes, especially if the patient continues to clinically deteriorate. Although an interfering agent is suspected to have caused the persistent elevation in vancomycin levels, it has not been identified, and we recommend further studies to explore agents that may have similar effects. In such cases where clinical deterioration occurs despite elevated drug levels, an alternative antibiotic must be immediately considered to prevent further morbidity and mortality, further emphasizing the importance of clinical judgment despite laboratory findings.
